# A Human Activity Recognition System Based on Dynamic Clustering of Skeleton Data

**DOI:** 10.3390/s17051100

**Published:** 2017-05-11

**Authors:** Alessandro Manzi, Paolo Dario, Filippo Cavallo

**Affiliations:** The BioRobotics Institute, Scuola Superiore Sant’Anna, Viale Rinaldo Piaggio, 34, 56026 Pontedera (PI), Italy; paolo.dario@santannapisa.it (P.D.); filippo.cavallo@santannapisa.it (F.C.)

**Keywords:** human activity recognition, clustering, x-means, SVM, SMO, skeleton data, depth camera, RGB-D camera, assisted living

## Abstract

Human activity recognition is an important area in computer vision, with its wide range of applications including ambient assisted living. In this paper, an activity recognition system based on skeleton data extracted from a depth camera is presented. The system makes use of machine learning techniques to classify the actions that are described with a set of a few basic postures. The training phase creates several models related to the number of clustered postures by means of a multiclass Support Vector Machine (SVM), trained with Sequential Minimal Optimization (SMO). The classification phase adopts the X-means algorithm to find the optimal number of clusters dynamically. The contribution of the paper is twofold. The first aim is to perform activity recognition employing features based on a small number of informative postures, extracted independently from each activity instance; secondly, it aims to assess the minimum number of frames needed for an adequate classification. The system is evaluated on two publicly available datasets, the Cornell Activity Dataset (CAD-60) and the Telecommunication Systems Team (TST) Fall detection dataset. The number of clusters needed to model each instance ranges from two to four elements. The proposed approach reaches excellent performances using only about 4 s of input data (~100 frames) and outperforms the state of the art when it uses approximately 500 frames on the CAD-60 dataset. The results are promising for the test in real context.

## 1. Introduction

Human activity recognition currently is an important and active research area in computer vision. Its goal is to automatically detect and analyze human activity information captured by various types of devices (e.g., color cameras or range sensors). This field of research can have broad applications, including security surveillance, human machine interaction, and home care for elderly people [1]. Although research in this area began in the early 1980s, it still presents challenges. In the past, color images from video cameras were used widely to perform activity recognition. Some methods that are based on human silhouettes use techniques such as hidden Markov model [2,3] or SVMs [4] to classify different postures. Other approaches employ the detection of scale-invariant spatiotemporal features [5]. In general, these solutions suffer from problems related to computational efficiency and robustness to illumination changes [6,7]. An alternative to 2D image sequences is represented by the use of 3D data acquired by marker-based motion capture or stereo camera systems [8].

Today, the advent of depth cameras (also known as RGB-D cameras) with relatively inexpensive costs and smaller sizes provides 3D data at reasonable frame rate resolution, leading to the emergence of many new developments in action recognition [9]. These particular devices, such as Microsoft Kinect and Asus Xtion, make available both color and depth information simultaneously. Moreover, specific software trackers that are able to extract human skeleton models from depth maps have been implemented [10,11]. These skeleton features can be used to develop technology and innovative services [12] in assisted living applications [13] that are not affected by environmental light variations while guaranteeing the user privacy much more than standard video cameras [14]. In literature, many authors focus on the use of multimodal features (i.e., combining color and depth information) [15,16]. Sung et al. [17] represent an activity as a set of subactivities, which is modeled using more than 700 features computing the Histogram of Oriented Gradient both on color images and on depth maps. A hierarchical maximum entropy Markov model is used to associate subactivities with a high-level activity. Wang et al. [18] introduce the concept of actionlet, which is a particular sequence of features that are termed local occupancy features. An activity is described as a combination of actionlets. Zhu et al. [19] employ several spatio-temporal interest point features extracted from depth maps in combination with skeleton joints to classify actions with an SVM. These methods can reach good results, but typically, their performances depend on the complexity of the background scene and the noise present in the depth data. Other approaches focus only on the use of human skeleton models to extract informative features to classify. Gaglio et al. [20] estimate the postures using a multiclass SVM and create an activity model with discrete HMM. Other works consider also trajectories of joints [21]. Some researchers focus on selection of the most informative joints to improve the classification results [22,23].

From the aforementioned literature overview on depth cameras, some authors focus on the use of multimodal features, while others exclusively rely on human skeleton data. The system presented in this work belongs to the latter case, and it is based on the concept of informative postures known as “key poses”. This concept has been introduced in [24] and extensively used in the literature [25,26]. Some authors identify key poses calculating the kinetic energy [27,28] to segment an activity in static and dynamic poses. In [29], an online classification method with a variable-length maximal entropy Markov model is performed based on the likelihood probabilities for recognizing continuous human actions. However, not all of the activities can be represented by alternating static and dynamic motions. Our approach is similar to [30], which uses clustering techniques to extrapolate the key poses, but conversely, our method represents an activity with a set of features based on a few basic informative postures.

The contribution of the paper is twofold. First of all, it aims at demonstrating that a small number of key poses is sufficient to describe and recognize a human activity. Differently from other works [23,30], our methods do not generate a bag of key poses for all the activities, but selects informative postures for each instance independently, ranging from two to four elements; secondly, this paper aims to evaluate the minimum number of frames to obtain an acceptable classification rate. The present work is a further research of our previous paper [31] that introduces the use of a dynamic clustering method during the classification phase. The idea is to extract during the classification phase and then on-the-fly, the optimal number of clusters of an input sequence. In this way, this method takes into account the fact that activities are more complex than others and also that the same action performed by different persons can be modeled with a number of clusters which are not essentially the same. Since we do not fix a priori the number of clusters, the developed system is able to perform a classification also on a subset of the input sequence without changing the implementation. As a consequence, it is possible to evaluate the minimum number of frames needed for the best classification.

In particular, the paper presents a human activity recognition system that uses skeleton data and machine learning techniques. An activity is represented with a few basic postures obtained with a clustering algorithm. The training process creates multiple models for each activity using a multiclass SVM, trained with Sequential Minimal Optimization (SMO). The classification phase processes the input sequence calculating the optimal number of clusters employing the X-means algorithm. Therefore, an action can be represented by a different number of centroids that is dynamically retrieved only during the classification. The system is trained on two publicly available datasets, the CAD-60, widely used for activity recognition, and the TST, a dataset created for ambient assisted living (AAL) applications. The performances outperform the state of the art on these datasets, making the system feasible for a real application.

The remainder of the paper is organized as follows. Section 2 details the developed system and the training and classification phases. The experimental results for two public datasets are presented in Section 3, while Section 4 concludes the paper.

## 2. System

The developed system implements a human activity recognition method using the 3D skeleton joint data extracted from a depth camera. The choice of excluding the image information is driven by the fact that the final system has to guarantee as much privacy as possible for the user. Another important aspect to consider is the reduction of the overall complexity due to the fewer number of features used in the computational steps. The basic idea of the developed system is to describe an activity using several sequences of a few basic informative postures. The extracted features are used in combination with machine learning techniques to correctly classify the action. This system is an improvement of the work already presented in [31]. The actual implementation differs from the previous one mainly in the use of a dynamic clustering method during the selection of the most informative postures for the classification phase of unseen data. The current system is based on the idea that, since actions can be very different, the optimal number of basic skeleton poses used to describe them can vary. For this reason, the system has a slightly different implementation concerning the training and the test phase (i.e., the classification). The first implementation concerns the training of several models, describing the whole set of activities, while the latter uses one of these models to infer the class to which the input sequence belongs. These two phases share most of the same software structure, with the exception that the task of the first one is to generate and save suitable activity models, while the second uses these models to perform the classification. One of the goals for the use of dynamic clustering is to be able to perform activity classification also on a subset of the input sequence to evaluate the minimum amount of frames needed for a correct classification. The remainder focuses on the development of these phases.

### 2.1. Feature Extraction

The feature extraction process involves three steps. First, the relevant skeleton features (i.e., spatial joints) are extracted from the depth camera device using a skeleton tracker [10]. Then, the data are clustered several times to describe the activities using different numbers of centroids representing basic and informative postures. Afterwards, a temporal sequence is built for each set of clusters and a sliding window is applied to create the activity feature.

#### 2.1.1. Skeleton Features

The spatial joints composing a human skeleton are extracted from the depth camera using a specific tracker. The model of a human varies according to the software and the adopted device. In particular, a human can be modeled with 15 [10] or 25 [32] joints. Each joint is described with three-dimensional Euclidean coordinates with respect to the sensor. These raw data cannot be used directly because they are dependent on the position of the human and the sensor and also on the subject dimensions, such as height and limb length. To manage these issues, the original reference frame is moved from the camera to the torso joint, and the joints are scaled with respect to the distance between the neck and the torso joint. This normalization step, adopted also in other works [20,27,30], yields data more independent with respect to the person’s specific size and to the position between the sensor and the user.

Formally, if we consider a skeleton with *N* joints, the skeleton feature vector f is defined as

(1) $$f=[\;j_{1},j_{2},\ldots ,j_{N-1}\;],$$

where each $j_{i}$ is the vector containing the 3D normalized coordinates of the *i*th joint $J_{i}$ detected by the sensor. Therefore, $j_{i}$ is defined as

(2) $$j_{i}=\frac{J_{i}-J_{0}}{∥J_{1}-J_{0}∥},\;i=1,2,...,N-1,$$

where $J_{0}$ and $J_{1}$ are the coordinates of the torso and the neck joint, respectively. These normalized skeleton features can be seen as a set of distance vectors related to the torso joint. The number of attributes of the feature vector f in Equation (1) is equal to 3(N-1). The experimental results of our previous work [31] show that the use of a reduced set of joints produces better classification performances. Hence, also for the present work, we use a feature vector with N=7, namely, the head, neck, hands, and feet, plus the torso as a reference. This restricted set of joints has shown itself to be the most discriminative for activity recognition, allowing reduction of complexity for the further steps of computation. As a consequence, a posture feature is made by 18 attributes. The next section describes how the most informative postures are selected from the entire activity sequence.

#### 2.1.2. Posture Selection

The idea of the whole system is to represent an activity using several sequences of a few basic postures. A posture is the feature vector f of Equation (1), i.e., the 3D coordinates of the joints composing the skeleton. The posture selection phase aims to select general and informative postures for each activity by means of a clustering technique. Hence, the goal is to group similar postures into certain clusters that share similar features. Nevertheless, some activities can be more complex than others, and the optimal number of clusters may vary according to this complexity. For this reason, conversely from [31], the system generates several models for each activity without determining the number of basic postures that describe an activity. A similar approach has been introduced in [23], which adopts a bag of key poses and dynamic time warping to calculate the fitness of their evolutionary algorithm. Therefore, the input sequence is clustered multiple times using a different number of clusters (*K*) to generate multiple samples representing the same activity. Technically, the K-means algorithm, introduced in [33] and developed in many variations [34,35], is applied several times to the input sequence, varying the desired number of *K* clusters. In Section 3, a description is provided of the procedure adopted to find a suitable range for the value of *K*.

In detail, given an activity composed by *M* posture features $\left[f_{1},f_{2},\ldots ,f_{M}\right]$, the K-means gives *k* clusters $\left[C_{1},C_{2},\ldots ,C_{k}\right]$, so as to minimize the intra-cluster sum of squares

(3) $$\underset{C}{arg min}\sum_{j=1}^{k}\sum_{f_{i}\in C_{j}}{∥f_{i}-\mu_{j}∥}^{2},$$

where $\mu_{j}$ is the mean value of the cluster $C_{j}$. At this stage, the set of posture features $f_{i}$ representing an activity sequence is replaced with the centroid to which the posture feature belongs. Hence, the centroids can be seen as the key poses of the activity.

#### 2.1.3. Activity Feature Generation

The activity feature generation step is the core of the system, and it is the same for the training and the classification phases. Its aim is to generate suitable features able to encode an activity. The input of this module, which is the output of the previous one (see Section 2.1.2), is a temporally ordered stream of the centroids representing the most important postures of the original skeleton sequence. The cardinality of this set is equal to the number of frames constituting the original action data. These features need to be reduced to increase the generality of the representation and also to lower its complexity. In addition, since different people perform the activities at different speeds, another important aspect to consider is the speed invariance of the new features. For this reason, the activity feature generation module considers only the transitions between key poses (i.e., transitions between centroids). Hence, the temporal sequence is now simplified to include only the transitions between clusters. This means that all the equal centroids that are consecutive in temporal order are discarded. This new compressed sequence provides a more compact representation of the activity and is also speed invariant. In other words, an action sequence is now encoded in a temporally ordered sequence of centroid transitions. Starting from this type of data, the idea is to exploit all the information contained in this sequence to represent a specific action in a way that is as general as possible using only a few basic postures. Let’s consider a person who is drinking a glass of water. We want our system to classify this action when the person is drinking in several gulps but also when the person begins to move his or her hand close to the mouth. Therefore, we generate *n*-tuple from the current sequence by means of a sliding window, creating a set of new instances to represent the specific activity. Consequently, the new instances are composed of a set of features with a size of 3L(N-1), where *L* is the length of the sliding window and *N* is the number of selected skeleton joints. Let’s take a simple example to clarify this concept. If an activity has three clusters, a possible compressed sequence can be

(4) $$A=[C_{1},C_{3},C_{2},C_{3},C_{2},C_{3},C_{2},C_{3},C_{2}].$$

**Figure 1:**
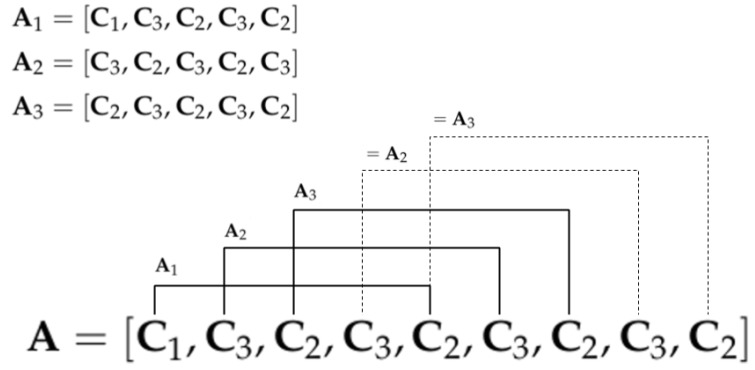
Example of activity feature instances using a sliding window of L=5 elements. Duplicates are discarded.

**Figure 2:**
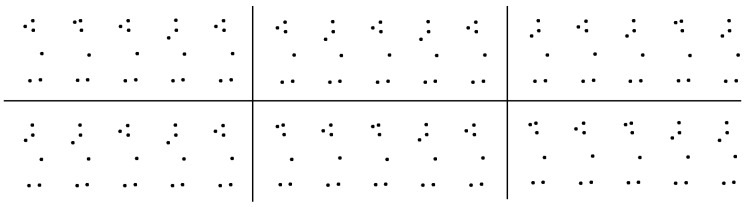
Subset example of activity feature instances using a window length equal to 5 and a skeleton of seven joints (torso omitted).

A sliding window with a size of L=5 elements produces three activity feature instances as depicted in Figure 1. Duplicates are not taken into account. The cardinality of the instances is related to the number of different transitions between different key poses. This means that actions that are repetitive during that time will have fewer feature instances than the ones with more variability between key poses. However, the newly generated instances are directly proportional to the window size in most of the cases. Figure 2 shows an example of six activity feature instances of the drinking action using L=5 and N=7 (torso is omitted). The output of this module is a new dataset containing the activity features, whose instances have a weight that is increased if the same tuple appears in the sequence. At the end of this step, each input activity is represented with several new activity features generated from the different sets of basic clusters.

#### 2.1.4. Dynamic Clustering

The dynamic clustering is employed only during the classification step. The core of this phase is to dynamically cluster the input sequence to use a different number of centroids to describe the activity, considering the heterogeneity of the action types. In addition, this method yields advantages when the system has to classify just a subset of the input sequence. The optimal number of clusters is obtained using the X-means algorithm [36], which is an optimized version of the K-means that does not need to know a priori the number of classes. It attempts to split the centers into regions and to select the number of clusters using a probabilistic scheme called Bayes Information Criterion. The X-means is much faster rather than running repeatedly K-means using different numbers of clusters, and it has proved to be less prone to local minima than its counterpart [37]. For each activity, the X-means is applied using the Euclidean distance function as a metric. It minimizes the intra-cluster sum of squares using the Formula (3).

### 2.2. Training Phase

**Figure 3:**
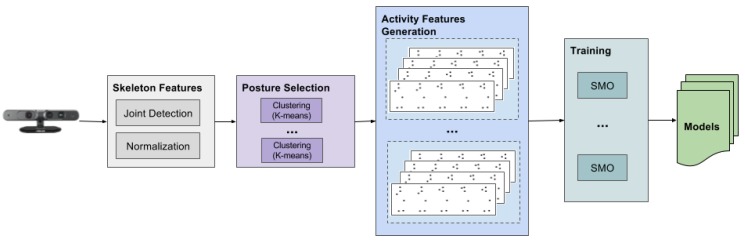
Software architecture of the training phase. The skeleton data are gathered from the depth camera, and the skeleton features are selected and normalized. Then, the input is clustered several times to find the informative postures for the sequence. The activity features are generated from the obtained basic postures, and, finally, a classifier is trained and relative models generated.

The models are generated training a classifier on the obtained activity features, creating one model for each set of clusters. The Sequential Minimal Optimization (SMO) [38] process is adopted to train a multiclass Support Vector Machine (SVM) [39] using the activity features obtained in the previous step (see Section 2.1.3). SVMs are supervised learning models used for binary classification to calculate the optimal hyperplane that separates two classes in the feature space. SMO makes use of improved internal structures and linear kernels to optimize the training phase. The multiclass SVM version is implemented by combining several binary SVMs using, in our case, a one-versus-one strategy [40]. At the end of this phase, each activity feature (i.e., each tuple) is associated with an activity and different models, related to the number *K* of clusters used in the posture selection phase (see Section 2.1.2). The whole training phase is depicted in Figure 3, while the pseudocode of the procedure is reported in Algorithm 1.

**Algorithm 1:** Pseudocode of the training phase.
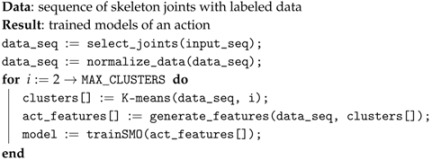


### 2.3. Classification Phase

**Figure 4:**
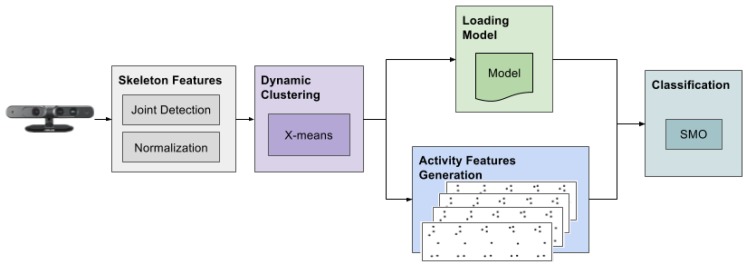
Software architecture of the testing phase. As for the training phase, the skeleton features are extracted from the depth camera. Then, the optimal number of clusters is calculated using the X-means algorithm. A classifier is applied using the previously trained model and the generated activity features.

The classification phase takes as input the activity features generated from the clustered data (see Section 2.1.3) and the trained model relative to the number of clusters *K* obtained from the dynamic clustering step (see Section 2.1.4). Each generated activity instance is classified with the same classifier as the training phase (Section 2.2), and each observed class is summed up. Hence, the final result is the class that has the greater value; therefore, it incorporates all of the classification results for each feature instance. The general architecture of the classification phase is depicted in Figure 4. It shares some software modules with the training phase, such as the Skeleton Features extraction and the generation of the Activity Features. It primarily differs in the clustering step, and, of course, it does not generate the models (i.e., it does not perform any training). The pseudocode of the classification phase is reported in Algorithm 2.
**Algorithm 2:** Pseudocode of the classification phase.**Data**: sequence of skeleton joints**Result**: actiondata_seq := select_joints(input_seq);data_seq := normalize_data(data_seq);clusters[] := Xmeans(data_seq);model := load_model(number_of_clusters);act_features[] := generate_features(data_seq, clusters[]);action := classifySMO(model,data_seq);

## 3. Results

The system is implemented in Java using the Weka library [41] (University of Waikato, Hamilton, New Zealand), which is an open source software containing a collection of machine learning algorithms for data mining tasks. We have tested our system on two publicly available datasets: the first one is the well-known Cornell Activity Dataset (CAD-60) [17] (Cornell University, Ithaca, New York, USA), widely used in activity recognition, while the second one is the fairly new TST dataset [42] (University of Marche, Ancona, Italy), specifically created for activities of daily living (ADL).

The software trackers used to build the datasets are different. Therefore, the skeleton of the first dataset is modeled with 15 joints (NiTE tracker), while the latter is modeled with 25 joints (Microsoft NUI). Several parameters need to be found to run the implemented system on the datasets. First, following the outcome of our previous work [31], we use a subset of the skeleton joints: specifically, the head, neck, hands, and feet. Hence, N=7, including the torso as a reference. Then, we perform several tests using a different value for the sliding window during the activity feature generation phase. In addition, since our goal is also to assess the performances on a subset of the input sequence, we conduct tests varying the input data by means of a selection window (with a 50% overlap) that splits the input sequence. The following sections address the results obtained for the two datasets.

### 3.1. CAD-60 Dataset

The Cornell Activity Dataset (CAD-60) focuses on realistic actions from daily life. It is collected using a depth camera and contains actions performed by four different human subjects: two males and two females. Three of the subjects use the right hand to perform actions, while one of them uses the left hand. It contains 12 types of actions: “talking on the phone”, “writing on whiteboard”, “drinking water”, “rinsing mouth with water”, “brushing teeth”, “wearing contact lenses”, “talking on couch”, “relaxing on couch”, “cooking (chopping)”, “cooking (stirring)”, “opening pill container”, and “working on computer”. The dataset contains RGB, depth, and skeleton data, with 15 joints available. Each subject performs the activity twice, so one sample contains two occurrences of the same activity. To be able to compare the results, we employ the same experimental settings as [17]. It consists of two cases: the “have seen” and “new person” setting. In the first case, the classification is done with the data of all four persons, splitting the data in half. The latter uses a leave-one-actor-out cross-validation approach for testing. This means that the classifier is trained on three of the four people and tested on the fourth. Since one person is left-handed, all the skeleton data are mirrored with respect to the sagittal plane of the skeleton. Conversely from [27], in which the right- and left-handed samples are trained and tested separately, our samples contains both original and mirrored data. As for the other works in the CAD dataset, the performance is reported in terms of the average precision and recall among all the activities according to the “new person” test case, plus the accuracy for sake of completeness.

To find the maximum number of clusters needed for the selection of the posture step, we run the X-means algorithm to determine the optimal number of clusters according to each activity, which is four. Therefore, the models created for this dataset range from a minimum of two to a maximum of four clusters. We conduct tests varying the length of the sliding window activity, ranging from five to 12. As a consequence, the lengths of the activity features have a minimum value of 90 and a maximum of 216, according to the window size. In addition, to assess the system on a subset of the input sequence, we also adopt a frame split window with 50% overlap and with the following length values: 100, 300, 500, and 700 frames. Table 1 reports the optimal number of clusters obtained with the X-means algorithm considering the number of analyzed frames. The performances for the “have seen” setting, as with other methods developed for the CAD-60, reaches 100% for all the samples. The outcome for the “new person” test case is much more interesting. The results show that a sliding activity window of 11 elements produces the best performances, confirming the outcome of our previous work [31]. Table 2 reports the performances of the dynamic clustering on CAD-60 related to the different frame subsets of the input sequence. The values of precision, recall, and accuracy reach 1 beginning from 500 frames. Considering that the frame rate of the samples in the dataset is ~25 Hz, the system needs approximately 20 s to classify all of the activities correctly. However, if we consider a frame split of 100 elements, we can note that the performances are still quite high. Therefore, we also report the confusion matrix of this case in Table 3. Looking at the classification results, we can note how all the activities are correctly classified using 100 frames, with the only exception the *open pill container*, which is misclassified with the *wearing contact lenses* for 44% of the cases. This means that, with only about 4 s of samples, the system already performs well. Table 4 reports the current six best results of the CAD-60 dataset, and our result outperforms the other works. However, it is worth noting that we are evaluating our system using a sliding window classification that differs from others which use frame to frame classification.

### 3.2. TST Dataset

The TST dataset (version 2) [42] is collected using the Microsoft Kinect v2 device (Microsoft, Redmond, Washington, USA). It is composed of ADL and Fall actions simulated by 11 volunteers. The people involved in the test are aged between 22 and 39, with different heights (1.62–1.97 m) and sizes. The actions performed by a single person are separated into two main groups: ADL and Fall. Each activity is repeated three times by each subject involved. The dataset provides eight actions and 264 sequences for a total of 46 k skeleton samples. Each person performs the following ADL movements: “sit on chair”, “walk and grasp an object”, “walk back and forth”, “lie down”, and the following Fall actions: “frontal fall and lying”, “backward fall and lying”, “side fall and lying”, “fall backward and sit”. We employ the same experimental settings using the aforementioned “new person” settings. Conversely from the CAD dataset, the lengths of the TST samples are much shorter: approximately 180 frames per action. Hence, we split the input sequence with an input split size of 30, 50, 60, and 100 frames with an overlap of 50%.

Applying the X-means algorithm, we obtain a maximum number of cluster of K=4, like the previous test. Therefore, also in this case, the number of the generated models ranges from a minimum of two to a maximum of four clusters. Table 5 reports the optimal number of clusters for each activity, considering the number of analyzed frames. From the results, we confirm our previous outcome that, for this dataset, the best sliding window length is 5. Table 6 reports the performances of the dynamic clustering related to the different values of the input window frame split. The best values are obtained using 100 frames, reaching 100% for the ADL actions and precision, recall, and accuracy greater than 90% for the Falling events. Looking at the classification results of the confusion matrix for the Fall events with 100 frames (see Table 7), only the *backward fall* is misclassified as a *frontal fall* in 25% of the cases.

## 4. Conclusions

This paper describes a human activity recognition system based on skeleton data gathered from a depth camera and on the use of machine learning techniques. Each activity is modeled using a different number of clusters that are extracted independently from activity instances, allowing for the use of far less human skeletons compared to other methods. In the training phase, several models are generated according to the different numbers of clusters. A multiclass SVM, trained with the SOM optimization, is used to create these models. During the classification step, the optimal number of key poses representing an activity is dynamically calculated employing the X-means clustering method. Depending on the input sequence and on the action, these numbers can vary, consequently leading to a dynamic generation of the clusters. As a consequence, since the number of clusters is not fixed a priori, the present work aims at assessing the classification performances for a subset of the input sequence to evaluate the minimum number of frames needed for a correct classification.

The system is tested on two publicly available datasets, the CAD-60 and the TST (version 2). The results point out that the developed model reaches excellent performances using approximately 4 s of input data (~100 frames), outperforming the state of the art when it uses approximately 500 frames on the CAD-60 dataset. The use of a small number of clusters adapted dynamically to the activity instances enhances the classification rate.

These encouraging results make it feasible to use the developed system in real environments. The adoption of skeleton features guarantees a higher level of user privacy compared to the standard video camera and ensures that the system is not affected by environmental light variations. However, the limitation of the skeleton features is that information about the surrounding objects is not provided. In fact, these could be exploited to model an action with objects. Moreover, the skeleton tracker software encounters difficulties in the presence of occlusions and prone positions. Another issue of the adopted classifier is that it is not able to handle unknown classes, i.e., it always tries to fit the data in one of the trained classes. Further study will be conducted to develop a real-time system for assistive scenarios exploiting the presented system.

## Figures and Tables

**Table 1:** Number of clusters obtained with the X-means algorithm considering a different input split frame size for each activity of the CAD-60.

Frame Split	100	300	500	700
	Number of Clusters			
talking on the phone	3	4	4	4
writing on whiteboard	2	2	2	2
drinking water	3	4	4	4
rinsing mouth with water	3	4	4	4
brushing teeth	4	4	4	4
wearing contact lenses	4	4	4	4
talking on couch	4	3	4	4
relaxing on couch	3	4	4	4
cooking (chopping)	2	4	4	4
cooking (stirring)	4	4	4	4
opening pill container	4	4	4	4
working on computer	2	2	2	4

**Table 2:** Overall precision, recall, and accuracy values using dynamic clustering and a sliding activity window of 11 elements on CAD-60.

Frame Split	“New Person”		
	Precision	Recall	Accuracy
100	0.963	0.958	0.984
300	0.950	0.958	0.986
500	1	1	1
700	1	1	1

**Table 3:**
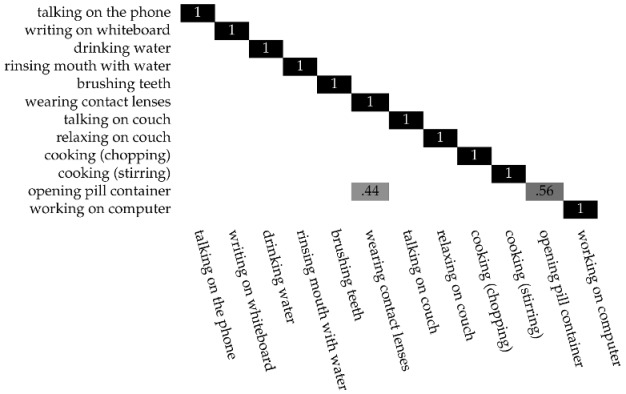
The confusion matrix of the “new person” test case using a sliding window activity size of 11 elements on CAD-60 with a window split of 100 frames.

**Table 4:** State of the art of precision and recall values (%) on CAD-60 dataset.

Algorithm	Precision	Recall
Zhu et al. [19]	93.2	84.6
Faria et al. [43]	91.1	91.9
Shan et al. [27]	93.8	94.5
Parisi et al. [44]	91.9	90.2
Cippitelli et al. [30]	93.9	93.5
Our First Method [31]	99.8	99.8
Current Method	100	100

**Table 5:** Number of clusters obtained with the X-means algorithm considering a different input split frame size for each activity of the TST dataset.

Category	Activity/Frame Split	Number of Clusters			
		30	50	60	100
	sit on chair	3	3	4	4
	walk and grasp	4	4	3	4
ADL	walk back and forth	3	3	3	3
	lie down	3	4	4	4
	frontal fall	3	3	3	4
	backward fall	2	3	4	2
Fall	side fall	3	4	4	4
	backward fall and sit	2	2	2	2

**Table 6:** Overall precision, recall, and accuracy values using dynamic clustering and a sliding activity window of five elements on TST.

Frame Split	ADL			Fall		
	Precision	Recall	Accuracy	Precision	Recall	Accuracy
30	0.956	0.911	0.927	0.687	0.516	0.527
50	0.953	0.906	0.921	0.767	0.667	0.667
60	1	1	1	0.826	0.819	0.805
100	1	1	1	0.937	0.950	0.933

**Table 7:**
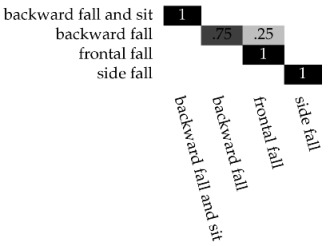
The confusion matrix of the TST Fall activity with 100 input frames.

## References

[1] Poppe R. (2010). A survey on vision-based human action recognition. Image Vis. Comput..

[2] Yamato J., Ohya J., Ishii K. Recognizing human action in time-sequential images using hidden markov model. Proceedings of the 1992 IEEE Computer Society Conference on Computer Vision and Pattern Recognition (CVPR’92).

[3] Kellokumpu V., Pietikäinen M., Heikkilä J. Human activity recognition using sequences of postures. Proceedings of the IAPR Conference on Machine Vision Applications (IAPR MVA 2005).

[4] Scholkopf B., Smola A.J. (2001). Learning with Kernels: Support Vector Machines, Regularization, Optimization, and Beyond.

[5] Willems G., Tuytelaars T., Van Gool L. An efficient dense and scale-invariant spatio-temporal interest point detector. Proceedings of the European Conference on Computer Vision.

[6] Aggarwal J.K., Ryoo M.S. (2011). Human activity analysis: A review. ACM Comput. Surv..

[7] Weinland D., Ronfard R., Boyer E. (2011). A survey of vision-based methods for action representation, segmentation and recognition. Comput. Vis. Image Underst..

[8] Argyriou V., Petrou M., Barsky S. (2010). Photometric stereo with an arbitrary number of illuminants. Comput. Vis. Image Underst..

[9] Aggarwal J.K., Xia L. (2014). Human activity recognition from 3d data: A review. Pattern Recognit. Lett..

[10] Shotton J., Sharp T., Kipman A., Fitzgibbon A., Finocchio M., Blake A., Cook M., Moore R. (2013). Real-time human pose recognition in parts from single depth images. Commun. ACM.

[11] Han J., Shao L., Xu D., Shotton J. (2013). Enhanced computer vision with microsoft kinect sensor: A review. IEEE Trans. Cybern..

[12] Turchetti G., Micera S., Cavallo F., Odetti L., Dario P. (2011). Technology and innovative services. IEEE Pulse.

[13] Cavallo F., Aquilano M., Bonaccorsi M., Mannari I., Carrozza M., Dario P. Multidisciplinary approach for developing a new robotic system for domiciliary assistance to elderly people. Proceedings of the 2011 Annual International Conference of the IEEE Engineering in Medicine and Biology Society.

[14] Padilla-López J.R., Chaaraoui A.A., Gu F., Flórez-Revuelta F. (2015). Visual privacy by context: Proposal and evaluation of a level-based visualisation scheme. Sensors.

[15] Ni B., Pei Y., Moulin P., Yan S. (2013). Multilevel depth and image fusion for human activity detection. IEEE Trans. Cybern..

[16] Ni B., Wang G., Moulin P. (2013). Rgbd-hudaact: A color-depth video database for human daily activity recognition. Consumer Depth Cameras for Computer Vision.

[17] Sung J., Ponce C., Selman B., Saxena A. Unstructured human activity detection from rgbd images. Proceedings of the 2012 IEEE International Conference on Robotics and Automation (ICRA).

[18] Wang J., Liu Z., Wu Y. (2014). Learning actionlet ensemble for 3D human action recognition. Human Action Recognition with Depth Cameras.

[19] Zhu Y., Chen W., Guo G. (2014). Evaluating spatiotemporal interest point features for depth-based action recognition. Image Vis. Comput..

[20] Gaglio S., Re G.L., Morana M. (2015). Human activity recognition process using 3D posture data. IEEE Trans. Hum.-Mach. Syst..

[21] Ding W., Liu K., Cheng F., Zhang J. (2015). STFC: Spatio-temporal feature chain for skeleton-based human action recognition. J. Vis. Commun. Image Represent..

[22] Jiang M., Kong J., Bebis G., Huo H. (2015). Informative joints based human action recognition using skeleton contexts. Signal Process. Image Commun..

[23] Chaaraoui A.A., Padilla-López J.R., Climent-Pérez P., Flórez-Revuelta F. (2014). Evolutionary joint selection to improve human action recognition with RGB-D devices. Expert Syst. Appl..

[24] Baysal S., Kurt M.C., Duygulu P. Recognizing human actions using key poses. Proceedings of the 2010 20th International Conference on Pattern Recognition (ICPR).

[25] Ballan L., Bertini M., Bimbo A.D., Seidenari L., Serra G. Effective Codebooks for human action categorization. Proceedings of the 2009 IEEE 12th International Conference on Computer Vision Workshops (ICCV Workshops).

[26] Raptis M., Sigal L. (2013). Poselet Key-Framing: A Model for Human Activity Recognition. Proceedings of the 2013 IEEE Conference on Computer Vision and Pattern Recognition (CVPR ’13).

[27] Shan J., Akella S. 3D human action segmentation and recognition using pose kinetic energy. Proceedings of the 2014 IEEE International Workshop on Advanced Robotics and its Social Impacts.

[28] Zhu G., Zhang L., Shen P., Song J., Zhi L., Yi K. Human action recognition using key poses and atomic motions. Proceedings of the 2015 IEEE International Conference on Robotics and Biomimetics (ROBIO).

[29] Zhu G., Zhang L., Shen P., Song J. (2016). An Online Continuous Human Action Recognition Algorithm Based on the Kinect Sensor. Sensors.

[30] Cippitelli E., Gasparrini S., Gambi E., Spinsante S. (2016). A Human Activity Recognition System Using Skeleton Data from RGBD Sensors. Comput. Intell. Neurosci..

[31] Manzi A., Cavallo F., Dario P., Hua G., Jégou H. (2016). A 3D Human Posture Approach for Activity Recognition Based on Depth Camera. Proceedings of the Computer Vision—ECCV 2016 Workshops.

[32] Microsoft Natural User Interface for Kinect. https://msdn.microsoft.com/en-us/library/hh855352.aspx.

[33] MacQueen J. (1967). Some methods for classification and analysis of multivariate observations. Proceedings of the Fifth Berkeley Symposium on Mathematical Statistics and Probability.

[34] Kanungo T., Mount D.M., Netanyahu N.S., Piatko C.D., Silverman R., Wu A.Y. (2002). An efficient k-means clustering algorithm: Analysis and implementation. IEEE Trans. Pattern Anal. Mach. Intell..

[35] Arthur D., Vassilvitskii S. K-means++: The advantages of carefull seeding. Proceedings of the Eighteenth Annual ACM-SIAM Symposium on Discrete Algorithms.

[36] Pelleg D., Moore A.W. X-means: Extending K-means with Efficient Estimation of the Number of Clusters. Proceedings of the Seventeenth International Conference on Machine Learning.

[37] Witten I.H., Frank E., Hall M.A. (2011). Data Mining: Practical Machine Learning Tools and Techniques.

[38] Platt J., Schoelkopf B., Burges C., Smola A. (1998). Fast Training of Support Vector Machines using Sequential Minimal Optimization. Advances in Kernel Methods—Support Vector Learning.

[39] Chang C.C., Lin C.J. (2011). LIBSVM: A library for support vector machines. ACM Trans. Intell. Syst. Technol..

[40] Hastie T., Tibshirani R., Jordan M.I., Kearns M.J., Solla S.A. (1998). Classification by Pairwise Coupling. Advances in Neural Information Processing Systems.

[41] Hall M., Frank E., Holmes G., Pfahringer B., Reutemann P., Witten I.H. (2009). The WEKA Data Mining Software: An Update. SIGKDD Explor. Newsl..

[42] Gasparrini S., Cippitelli E., Gambi E., Spinsante S., Wåhslén J., Orhan I., Lindh T. (2016). Proposal and experimental evaluation of fall detection solution based on wearable and depth data fusion. ICT Innovations 2015.

[43] Faria D.R., Premebida C., Nunes U. A probabilistic approach for human everyday activities recognition using body motion from RGB-D images. Proceedings of the 23rd IEEE International Symposium on Robot and Human Interactive Communication.

[44] Parisi G.I., Weber C., Wermter S. (2015). Self-Organizing Neural Integration of Pose-Motion Features for Human Action Recognition. Front. Neurorobot..

